# An Innovative High-Tech Acupuncture Product: SXDZ-100 Nerve Muscle Stimulator, Its Theoretical Basis, Design, and Application

**DOI:** 10.1155/2012/626395

**Published:** 2012-02-09

**Authors:** Xinyan Gao, Peijing Rong, Liang Li, Wei He, Hui Ben, Bing Zhu

**Affiliations:** Department of Physiology, Institute of Acupuncture and Moxibustion, China Academy of Chinese Medical Sciences, Beijing 100700, China

## Abstract

We introduce the theoretical basis, design, and application of a patented innovative high-tech product, SXDZ-100 nerve and muscle stimulator. This product is featured with a built-in chip containing transcoding information from different acupuncture manipulation collected from the wide dynamic neurons (WDR) in the spinal dorsal horn in animal experiments, which is bioinformation feedback therapy. The discharges of WDR neurons excited by different manipulations are analyzed using chaos theory in this study. It combines the advantages of manual acupuncture (MA) like no receptor adaptation and treatment individualization and that of electroacupuncture (EA) such as relatively low stimulation intensity and good quantification and thus makes it more effective than common stimulators in acupuncture clinic.

## 1. Introduction

**Figure 1:**
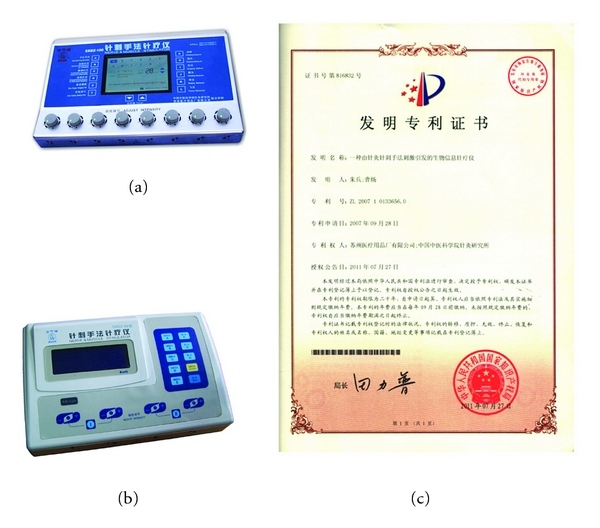
Introduction of SXDZ-100 and SXDZ-50. (a) SXDZ-100 nerve and muscle stimulator; (b) SXDZ-50 nerve and muscle stimulator; (c) certification of invention patent from State Intellectual Property Office of the People's Republic of China. SXDZ-100 is of bigger size and more expensive than SXDZ-50.

Acupuncture is a somatic stimulation therapy. Needling signal is a spatiotemporal grouped sequence of input neuroinformation [1]. Previous reports demonstrate that acupuncture is an effective approach which regulates the human body through encoding external stimulation [2]. Acupuncture manipulation, as one of the major components of acupuncture, is an essential skill for clinic practitioners. Previous studies show that the effect of manipulation of manual acupuncture (MA) is better than that of electroacupuncture (EA) with fixed parameters [3, 4], whereas others still think that EA is more effective, indicating that each stimulation refers to different disease states [5, 6]. However, MA is not easy to be explained and learnt by practitioners. In addition, to ensure the comparability among researchers or subjects in acupuncture research, EA is usually selected by investigators because of the well-quantified stimulation parameters [7, 8]. The problem is that the EA instruments with fixed pulses by settled intensity and frequency are easily adapted to [9]. Adaptation also occurs in the other sensory receptors [10]. EA stimulates mechanoreceptors that respond to mechanical pressure or distortion. This response to a continuous stable stimulation with fixed frequency and intensity will soon be adapted to and lead to an attenuation of the effect of EA in clinic. Thus, it is necessary to develop a novel EA instrument which can scientifically combine acupuncture manipulation and electric stimulation but avoid the adaption issue of EA simultaneously. Through combining chaos and acupuncture manipulation theories, we developed this patented instrument SXDZ-100 nerve and muscle stimulator (patent no. 200710133656.0, State Intellectual Property Office of the People's Republic of China), manufactured by Suzhou Hua Tuo Medical Instruments Co., Ltd., and applied it in clinic and basic research (Figures 1(a), 1(b), and 1(c)). Here, we would like to introduce our first findings. 

## 2. Theoretical Basis

In mammalians, there exist commonly known peripheral sensory fibers as A*α*, A*β*, and A*δ*. Nociceptive stimulation excites C fiber receptors, whereas nonnociceptive stimulation excites A*α*- and A*β*-fiber endings [11]. Wide dynamic range (WDR) neurons in lamina IV–VI of spinal dorsal horn, especially lamina V, respond to both nociceptive and nonnociceptive stimuli of any kind (thermal, chemical, and mechanical) imposed on the middle of the receptive field. This response is intensity dependent [12].

Acupoints are usually some excitable muscle or skin nerve complexes with high density of nerve endings, and the distribution of receptive fields of A*α*, A*β*, A*δ*, and C fibers is closely associated with the acupoints both in the skin and muscles [13]. Receptors excited by acupuncture or EA are mechanoreceptors and polymodal receptors [14]. Needling sensation receptors include touch unit, pressure unit, muscle spindles, or Golgi tendon receptor unit according to the location of the acupoint, and the receptors and the afferent fibers of the acupoint play a critical role in forming and maintaining the needling sensations [15]. Studies about the relationship between acupuncture manipulations and responsive discharges of deep receptors recorded in medial gastrocnemius nerve in rabbits demonstrated that every type of deep receptor can react to any manipulation, and that the discharge patterns of different receptors are alike when stimulated with the same manipulation, while different manipulations acting on the same receptor can induce different discharge patterns [16].

EA and MA are two different stimulations. During EA, repeated external currents or voltages are delivered to acupoints via the needles. Currents which are intense enough to excite A*β* and part of A*δ* fibers can produce an analgesic effect [17]. MA is the insertion of an acupuncture needle into an acupoint followed by different manual technique stimulations, and all types of afferent fibers (A*β*, A*δ*, and C) are activated [17]. Excited afferent fibers and evoked sensations are different between EA and MA. A*β* fibers are mainly excited, and numb sensation is produced by EA, whereas for MA, A*δ* fibers are excited, and sour and distending sensations are thus produced [15]. To obtain the same effect induced by MA, a certain amount of intensity will be requisite for EA [18].

De qi is the sensation following acupuncture needle placement and subsequent manipulation of the needle, which is important for treatment efficacy [19]. The complex pattern of sensations in the de qi response involves a wide spectrum of myelinated and unmyelinated nerve fibers, particularly the slower conducting fibers in the tendinomuscular layers [20]. De qi sensation was reported qualitatively and quantitatively different between manual and electrical stimulation; the most predominant de qi sensation with electrical stimulation appears to be tingling in nature. However, in manual stimulation, an aching sensation appeared to be the most predominant de qi sensation, followed by sharp pain and tingling sensations [21]. It is hypothesized that de qi is more easily obtained by a stimulation with combined acupuncture manipulations and electric stimulation in the present study. Pacinian corpuscles, for example, are pressure receptors located in the skin and various internal organs. Each pacinian corpuscle is a nerve ending of a sensory neuron. Mechanical pressure of varying strength and frequency induces corpuscle deformation, which creates a generator potential in the sensory neuron arising within it [22]. While reaching its threshold, generator potential induces action potentials, also called nerve impulses. Nerve impulses or action potentials are formed by pressure-sensitive sodium channels at the first node of Ranvier, the first node of the myelinated sensory neuron. The magnitude of the stimulus is encoded by the frequency of impulses generated in the neurons. The more massive or rapid the deformation of a single receptor corpuscle, the higher the frequency of nerve impulses generated in its neuron. This information is encoded by the frequency of impulses since a bigger or faster deformation induces a higher impulse frequency. Action potentials are formed when the skin is rapidly distorted but not when pressure is continuous. Therefore, with continuous pressure, the frequency of action potentials decreases quickly and stops soon. This is the phenomenon of receptor adaptation [10, 23]. The evidence shows that there exist four receptors and afferents responding differently to twisting and lifting-inserting manipulation, and both manipulations stimulate adaptive receptors [24].

It has been found in our study that the 12 most common acupuncture manipulations, including 6 monotypes and 6 multitypes, have individualized group bioinformation encodings. Receptor adaptation, featured with decaying neuronal discharges, existed during EA, but it was not found during MA stimulation [9]. Also it was found that different manipulations have different analgesic effects on somatic acute pain, inflammatory pain, and visceral pain, allowing the possibility for the practitioner to select the optimal stimulation to meet the need of individualized therapy [25].

In general, the monotype manipulations include twirling, handle waggling, handle scraping, trembling, handle flicking, and handle flying, and multitype manipulations include twirling supplementation and draining, lifting-thrusting supplementation and draining, quick-slow supplementation and draining, neutral supplementation and draining, blue dragon wagging tail, and dark tortoise seeking hole [26]. Acupuncture manipulations evoke afferent pulses as various electrical signal time series, called discharges in spinal dorsal horn neurons, which can be analyzed by nonlinear dynamics method to determine the optimal embedding dimension. The time series are considered chaotic by plotting the attractor, qualitatively. The largest Lyapunov exponents (LLEs) are computed based on the Takers' embedding theorem. The LLEs of the time series are positive and obviously vary in different acupuncture manipulations [27, 28].

**Figure 2:**
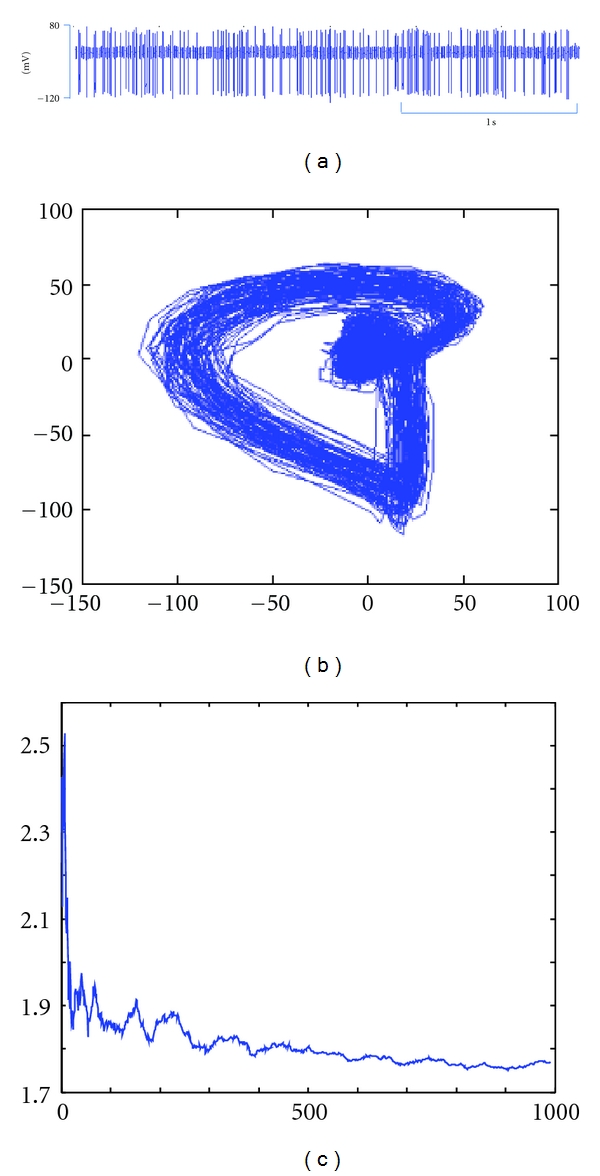
Chaos analysis for the signal recorded in the spinal dorsal horn neuron evoked by manual manipulation of neutral supplementation and draining on ST 36. (a) Time series of neuronal discharge; (b) the reconstructed attractors; (c) the LLE (1.7691).

**Figure 3:**
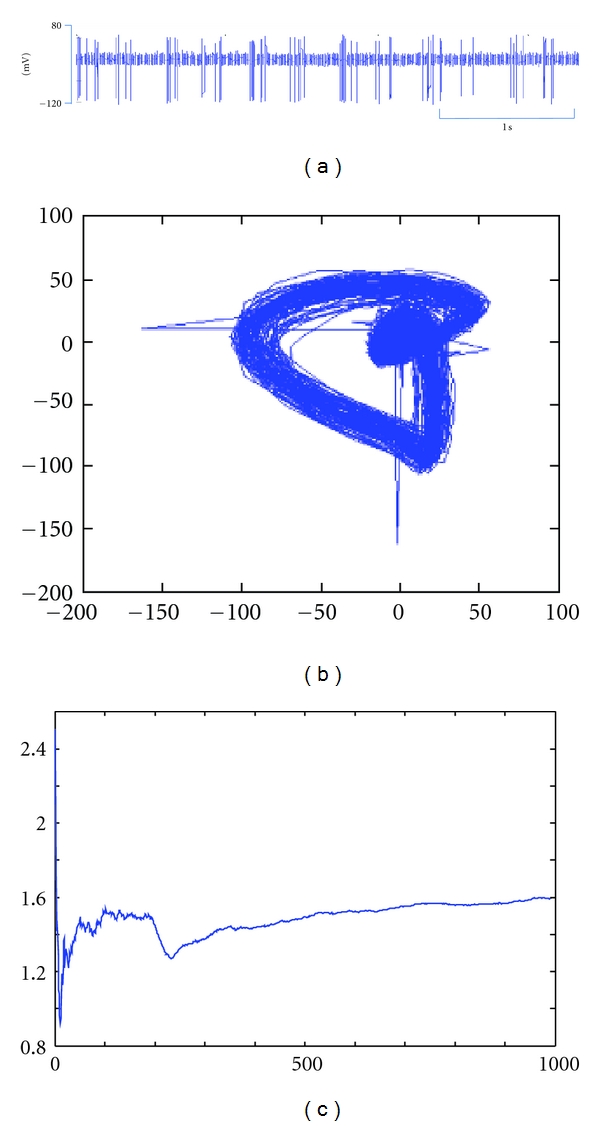
Chaos analysis for the signal recorded in the spinal dorsal horn neuron evoked by manual manipulation of blue dragon wagging tail on ST 36. (a) Time series of neuronal discharge; (b) the reconstructed attractors; (c) the LLE (1.5934).

The research concerning chaos theory has been made in many related fields such as power system and biomedical engineering as well as applications to the human brain and heart [29–31]. In the 1980s, Japanese investigators studied the repetitive firing of the action potential in squid giant axons stimulated by sinusoidal current and found various motions including periodic, quasiperiodic, and chaotic through theoretical computations [32, 33]. In our previous work, we successfully analyzed each time series of neuronal discharge in the dorsal horn neurons evoked by twisting, reinforcing-reducing by slow-quick needling, and reinforcing-reducing by lifting-inserting manipulations on ST36 using chaos theory [28]. In this paper, the time series as neuronal discharge signals of two manipulations as mild reinforcing-reducing and blue dragon wagging tail were analyzed also using chaos for the attractors of the reconstructed phase space and as examples of LLE. Of these two manipulations, mild reinforcing-reducing is easier than blue dragon wagging tail to be performed on patients (Figures 2 and 3). Therefore, this time series information of each manipulation is encoded in the built-in chips in SXDZ-100, as one of the most important steps. 

## 3. Design

**Figure 4:**
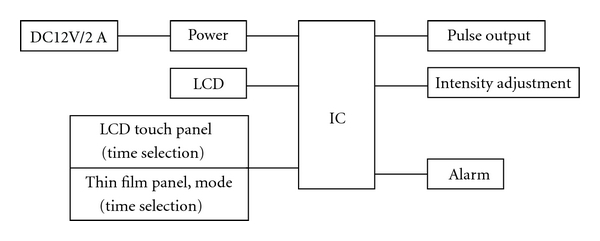
Design diagram of SXDZ-100. LCD: liquid crystal display; DC: direct current; IC: integrated circuit.

Based on the above theory, we designed the mechanism diagram of SXDZ-100 as seen in Figure 4. In this diagram, the chip is the core of the instrument. The built-in chips of SXDZ-100 contain the time series information of each manipulation. The pulses generated by SXDZ-100 are not just simply external currents, but a kind of encoded currents containing neurobiological messages collected from the human body, in accordance with the concept of bioinformation feedback therapy. Due to the built-in bioinformation group encoding system, SXDZ-100 is significantly different from commonly used electronic pulse generators. Its output is a continuous multivariate waveform which may not be easily adapted to by cutaneous and intramuscular receptors. It combines the advantages of both MA with uneasily adapted manual stimulation and EA with comparatively low stimulation intensity required. We designed and developed SXDZ-100 by selecting characteristic manipulations from numerous acupuncturists and acupuncture academicians. The manual skill signals between fingers of acupuncture specialists are cloned in spinal WDR neurons where decoding of the afferent stimulation signals happens through laboratory approach. The electronic signals produced by the built-in chip containing the afferent signal are not simple external currents, but neuroinformation encoding currents, meeting the needs of bio-information feedback therapy. Therefore, its output currents on needles mimic manual techniques. Figure 4 shows the circuit diagram of SXDZ-100.

## 4. Application and Discussion

SXDZ-100 has been widely utilized in acupuncture clinic. It has been reported that SXDZ-100 has a higher cure rate than the G6805 electric stimulator did for facial paralysis, indicating that it is a promising high-tech product and can be widely used and would be able to substitute old style electric stimulators in acupuncture clinic [34]. In acupuncture research, SXDZ-100 can also be used as a transcoding stimulator in the research whose common EA stimulator is used and possesses both the advantages of MA and EA. Considering wider applicability and lower cost, we also designed and developed a smaller version, the SXD-50 nerve and muscle stimulator, which is easier to operate and suitable for home use (Figure 1(b)).

Chaos analysis is a powerful nonlinear dynamic method enabling the extraction of characteristic quantities. It has been found that the phase space of the time series corresponding to the electrical activity of neurons is embedding parameters, and that the largest Lyapunov exponents are calculated and turned out to be positive, based on which, it is concluded that the neuronal signals are chaotic [28]. In this patented SXDZ-100 stimulator, the skillful manipulation signals produced by the fingers of acupuncture specialists as reflected in spinal WDR neurons are cloned. Thus, currents produced from the built-in chip containing the afferent signal codes are not simple biphasic currents, but neuro-information encoded currents, meeting the needs of bio-information feedback therapy. Acupuncture signal may be input into the central nervous system for final integration and organization [35]. In the present study, firings were recorded in the WDR neurons rather than those of the peripheral nerve fibers. Analysis of the input signal encoding of acupuncture helps us to understand the acupuncture information quantification and processing well.

Combined study of the acupuncture mechanism and encoding and the organization of neuro-information will definitely propel the profound understanding of both. Acupuncture manipulations are essential skills for practitioners in clinic, however, not easy to be expressed and learnt by successors. Notably, the effect of different manipulation of MA is better than EA with fixed parameters. However, in acupuncture research, to ensure the comparability among researchers and subjects, EA is mostly selected as the stimulation parameters can easily be quantified by stimulator setting. Although the discrepancy between basic research and clinical practice is obvious, it provides ideas for the invention of a novel electronic stimulator. On the other hand, studies about the mechanism of acupuncture and the improvement of the effect of EA have been conducted nationally and internationally. It is a promising trend to seek out a product with features as labor saving, substituting for MA with long-time needle manipulation, and easily set and assessed as electroacupuncture.

SXDZ-100 is an original scientific research product. It is a more significant innovation of Traditional Chinese Medicine acupuncture combined with electronic stimulation therapy. Compared to other therapeutic instruments both in the national and international markets, SXDZ-100 possesses several major advantages: (1) high-tech: it has built-in chips containing encoded group bio-information from different acupuncture manipulations and provides individualized EA stimulation with varied modes and intensities; (2) easy to operate: it can be easily operated by selecting solidified acupuncture manipulation module from specialists, and by tuning output intensity according to patients' constitution; (3) adjustable parameters: parameter is optional within the ranges ≤120 Hz, ≤300 *μ*s, and ≤70 V according to different manipulations; (4) endorsement of International Electrotechnical Commission (IEC) and Medical Device Directive (MDD): the circuit board and the whole machine are measured rigidly according to IEC- IEC60601-1, IEC60601-1-2 and IEC60601-2-10 of the US, and the European MDD is also executed to ensure that the criteria of SXDZ-100 are higher than the current national one, such as high- and low-temperature circulation, vibration and shocking, and electronic neutrality, electricity and construction safety, electromagnetic compatibility.

Supported by the above qualification, superiorities of SXDZ-100 are distinct compared to products like both EA apparatus and transcutaneous electrical stimulation (TENS) in the national and international markets. Firstly, merged functions of EA and MA perfectly lead to an innovation and development of both through combining the advantage of both MA with uneasily adapted manual stimulation and EA with comparatively low stimulation intensity required. Its output currents on needles mimic manual techniques well. SXDZ-100 with proprietary intellectual property rights in China is a novel product in Traditional Chinese Medicine acupuncture field. Secondly, SXDZ-100 has fixed stimulation module set by group bioinformation encoding and can be tuned individually to avoid the adaptation of the body. Thirdly, the output of SXDZ-100 is bipolar positive and negative currents. Ideally, bio-electronic stimulation should not be net direct current (DC), and it will be accessible to introduce train, symmetric current, or asymmetric countercurrent, which is another innovative point of SXDZ-100.
